# Real-World Effectiveness of Cisplatin, 5-Fluorouracil, and Pembrolizumab Combination Therapy for Unresectable or Recurrent Esophageal Cancer

**DOI:** 10.3390/cancers17193202

**Published:** 2025-10-01

**Authors:** Yu Ueta, Masanobu Nakajima, Masaki Yoshimatsu, Takahiro Ochiai, Shuhei Takise, Junki Fujita, Masatoshi Nakagawa, Shinji Morita, Kazuyuki Kojima

**Affiliations:** 1 Department of Surgical Oncology, Dokkyo Medical University Graduate School of Medicine, 880 Kitakobayashi, Mibu, Shimotsuga-gun, Tochigi 321-0293, Japan; u-yuu19@dokkyomed.ac.jp (Y.U.); my-1157@dokkyomed.ac.jp (M.Y.); 2 Department of Upper Gastrointestinal Surgery, Dokkyo Medical University, 880 Kitakobayashi, Mibu, Shimotsuga-gun, Tochigi 321-0293, Japan; t-ochiai213@dokkyomed.ac.jp (T.O.); s-takise@dokkyomed.ac.jp (S.T.); j-fujita@dokkyomed.ac.jp (J.F.); mnaksrg@dokkyomed.ac.jp (M.N.); shmorita@dokkyomed.ac.jp (S.M.); kojima-k@dokkyomed.ac.jp (K.K.)

**Keywords:** cisplatin, 5-fluorouracil, pembrolizumab, esophageal cancer

## Abstract

**Simple Summary:**

Esophageal cancer is one of the most aggressive cancers worldwide, and many patients are diagnosed at an advanced stage where surgery is not possible. A combination of cisplatin, 5-fluorouracil, and pembrolizumab has recently become a standard first-line treatment for unresectable or recurrent esophageal cancer, but many patients in real-world practice are elderly or have impaired renal function, which makes full-dose chemotherapy difficult to tolerate. In this study, we evaluated the outcomes of patients treated with this combination therapy at our institution, with particular attention to those who started treatment with reduced doses. We found that dose reduction did not compromise the effectiveness of treatment and was associated with fewer severe side effects, allowing patients to continue therapy for longer periods. These findings suggest that careful dose adjustment may help provide safe and effective treatment options for vulnerable patients in clinical practice.

**Abstract:**

Background: Immune checkpoint inhibitors (ICIs) such as pembrolizumab (Pem) have demonstrated clinical benefits in esophageal cancer. Cisplatin, 5-fluorouracil, and Pem combination (CF plus Pem) has emerged as a promising first-line regimen. However, dose reduction of cytotoxic agents is necessary in real-world practice in patients with advanced age and/or renal dysfunction. This study aimed to evaluate the real-world effectiveness and safety of CF plus Pem therapy and assess survival outcomes based on the initial dose intensity. Methods: We retrospectively analyzed patients with unresectable or recurrent esophageal cancer who received CF plus Pem between February 2022 and February 2025. Clinical data, including patient characteristics, treatment details, tumor response, adverse events, and survival outcomes, were collected and analyzed. Results: We included 54 patients (median age, 72.5 years; 74.1% male). The initial CF dose was reduced in 55.6% of the patients. The overall response and disease control rates were 55.6% and 81.5%, respectively. The median overall survival (OS) and progression-free survival (PFS) were 18.6 and 6.5 months, respectively, with no significant differences observed among groups based on dose reduction, age, or change in treatment interval. Grade ≥ 3 adverse events occurred in 16.7% of patients, with fewer events and higher treatment continuity in the dose-reduction group. Conclusions: Thus, CF plus Pem therapy is effective and tolerable in real-world settings. Initial dose reduction does not compromise survival and supports individualized dosing strategies for esophageal cancer treatment.

## 1. Introduction

Esophageal cancer is one of the most lethal gastrointestinal malignancies worldwide. The GLOBOCAN 2022 data indicated approximately 511,054 new cases and 445,391 deaths, making it the seventh leading cause of cancer-related mortality [1]. In Japan, approximately 24,000 new cases of esophageal cancer are reported annually, and this number is projected to increase further [2]. Patients tend to be diagnosed at advanced stages (III or IV), which significantly limits the possibility of curative treatment [3]. Cisplatin and 5-fluorouracil (CF) therapy has been the standard first-line treatment for unresectable or recurrent esophageal cancer, with reported response rates of approximately 30% and median overall survival (OS) ranging from 6.6 to 9.5 months [4,5,6,7,8,9]. Immune checkpoint inhibitors (ICIs) are now internationally recognized as a new standard of care in various malignancies including esophageal cancer owing to their superior efficacy [10,11,12,13]. The KEYNOTE-590 trial compared cisplatin, 5-fluorouracil (CF), and pembrolizumab (Pem) (CF plus Pem) with CF plus placebo. The addition of Pem significantly prolongs OS and progression-free survival (PFS), establishing CF plus Pem therapy as the global standard [13]. However, in clinical practice, CF regimens often require dose reduction in patients with advanced age, renal dysfunction, or poor performance status. Despite their common occurrence, the effects of these modifications on the treatment outcomes have not been sufficiently studied. This is particularly relevant in real-world settings where patients are often older and present with multiple comorbidities. This retrospective study aimed to evaluate the effect of dose modifications based on age and renal function on the efficacy and safety of combined CF and Pem therapy. We focused on the OS, PFS, response rates, and adverse events in patients with unresectable or recurrent esophageal cancer.

## 2. Materials and Methods

### 2.1. Study Design and Patients

In our institution, the treatment strategy is determined according to disease stage and resectability. For patients with Stage IVA disease, surgery aiming for R0 resection is performed when resection is feasible, such as in T4a cases with invasion to the lung or pleura. For unresectable disease, including T4b cases with invasion to the aorta, trachea, or bronchi, definitive chemoradiotherapy (CRT) is indicated. In patients with extensive lymph node metastases (N3), systemic therapy with ICI plus chemotherapy is administered. For Stage IVB disease, the principle of treatment is systemic therapy, with ICI plus chemotherapy being the standard approach. In addition, palliative treatments are considered when appropriate; for example, in cases of dysphagia, palliative radiotherapy (RT) or esophageal stent placement may be employed. For recurrent disease, when local therapy is feasible, chemotherapy is combined with surgery or radiotherapy, whereas when local therapy is not feasible, systemic therapy with ICI plus chemotherapy is selected.

This retrospective observational study was conducted at a single institution. We included patients with unresectable or recurrent esophageal cancer who received CF plus Pem therapy between February 2022 and February 2025. Patients with incomplete clinical data and those with a short observation period were excluded from this study. Patients with comorbidities who were able to continue treatment were included. The inclusion criteria were (i) histologically confirmed unresectable or recurrent esophageal cancer, and (ii) availability of sufficient clinical and follow-up data. The exclusion criteria were as follows: (i) patients with incomplete or missing clinical data, and (ii) patients with a short observation period.

This study was conducted in accordance with the principles of the Declaration of Helsinki and was approved by the Medical Ethics Committee of Dokkyo Medical University (approval number: R-96-1J). Written informed consent for treatment was obtained from all patients. For the use of clinical data, the requirement for additional individual consent was waived owing to the retrospective design, with an opt-out option available on the institutional website.

### 2.2. Data Collection Items

Clinical data were extracted from electronic medical records and imaging studies. Variables included age, sex, Eastern Cooperative Oncology Group performance status (PS), combined positive score (CPS) of programmed death-ligand 1 (PD-L1), estimated glomerular filtration rate (eGFR), and comorbidities. Tumor characteristics, including location, histological type, macroscopic appearance, clinical stage, and metastasis, were classified according to the Union for International Cancer Control (UICC) tumor, node, and metastasis (TNM) classification (8th edition). For patients with recurrence (*n* = 22), the T, N, M, and stage classifications refer to the clinical stage before the initial therapy, not at the time of recurrence.

The treatment-related data included the number of cycles, initial CF dose (percentage of the standard dose), reasons for dose reduction, and post-treatment course. Treatment response was assessed using the Response Evaluation Criteria in Solid Tumors (RECIST 1.1): complete response (CR), partial response (PR), stable disease (SD), or progressive disease (PD). Esophagogastroduodenoscopic (EGD) findings were reviewed. Safety was evaluated according to the Common Terminology Criteria for Adverse Events (CTCAE, version 5.0), including the incidence and grade of immune-related adverse events (irAEs).

### 2.3. Treatment Protocol

The standard regimen consisted of cisplatin (80 mg/m^2^ on day 1), 5-fluorouracil (800 mg/m^2^ on days 1–5), and Pem (200 mg/body on day 1), repeated every three weeks. Dose reductions of CF were considered in patients with impaired renal function, advanced age, or poor performance status. Specifically, in cases with creatinine clearance (Ccr) < 60 mL/min or eGFR < 60 mL/min/1.73 m², the initial dose was generally reduced to ≤80%. For patients with Ccr < 50 mL/min, the dose was further adjusted to 50–70%, and in those with a solitary kidney or severe renal impairment, the dose was reduced to approximately 50%. In elderly patients (≥75 years), the initial dose was reduced to 80–90% when the PS was 0–1, and to 50–70% in those with PS ≥ 2 or significant comorbidities. In patients with poor PS (≥2), the initial dose was usually reduced to 50–70% to minimize the risk of adverse events. In practice, the reduced-dose cohort consisted of two groups: 50% and 70%. Additional dose modifications were made on an individual basis, taking into account comorbid conditions (e.g., cardiac disease, sequelae of cerebrovascular disorders) or prior treatment-related toxicities. However, the Pem dosage was not reduced. If adverse events persisted, the treatment interval was extended to four weeks. The treatment was continued until disease progression or unacceptable toxicity was observed. The tumor response was assessed every 8 weeks in the first year and every 12 weeks thereafter.

### 2.4. Evaluation Criteria

The efficacy was assessed using the overall response rate (ORR [CR + PR]) and disease control rate (DCR [CR + PR + SD]). OS and PFS were measured from treatment initiation to death and cancer progression, respectively. Additionally, we evaluated the frequency and severity of treatment-related adverse events according to CTCAE version 5.0. Associations between initial dose intensity and outcomes were also examined.

### 2.5. Statistical Analyess

All analyses were performed using the Easy R software (EZR, version 1.55). Continuous variables are expressed as medians (range), and categorical variables are expressed as proportions. Chi-squared or Fisher’s exact tests were used for categorical comparisons. OS and PFS were estimated using the Kaplan–Meier method and compared using the log-rank test. Statistical significance was set at *p* < 0.05.

## 3. Results

### 3.1. Characteristics of the Patients

A total of 54 patients with unresectable or recurrent esophageal cancer who received CF plus Pem therapy between February 2022 and February 2025 were enrolled in this study. Their median age was 72.5 years (range, 44–91 years), and 40 patients (74.1%) were male. Overall, 75.9% of patients had PS of 0–1. CPS was examined in 23 patients: CPS ≥ 10 in 16 cases (69.6%), CPS < 10 in 7 cases (30.4%), and not assessed in 31 cases. Renal dysfunction, defined as eGFR < 60 mL/min/1.73 m^2^, was present in 15 patients (27.8%). In addition, 26 patients (48.1%) had creatinine clearance (Ccr) < 60 mL/min, including 15 patients (27.8%) with Ccr < 50 mL/min (Table 1). The most frequent tumor location was the middle thoracic esophagus; 53 patients had squamous cell carcinoma. The most common metastatic sites were the extra-regional lymph nodes, followed by the lungs and the liver.

### 3.2. Dose Intensity of Cisplatin and 5-Fluorouracil and Modification of the Treatment Interval

The initial CF dose was 100% in 24 patients (44.4%), 90% in 2 (3.7%), 80% in 9 (16.7%), and <80% in 19 (35.2%). Dose reduction was performed in patients with renal dysfunction (*n* = 24), advanced age (*n* = 14), or poor PS (*n* = 13). The treatment interval was extended to 4 weeks in 32 patients (59.3%) based on adverse events or PS during the first cycle, or if adverse events persisted thereafter (Table 2).

### 3.3. Treatment Outcomes

The ORR was 55.6%, including five cases of CR and 25 cases of PR. The DCR was 81.5%. In the lower dose group (initial CF dose < 80%), the ORR was 63.2% (CR, 2 cases; PR, 10 cases) and the DCR was 89.4%, with no significant difference compared to the non-reduction group. In older patients (aged ≥75 years), the ORR was 61.9% (CR, 3 cases; PR, 10 cases) and the DCR was 90.5% (Table 3). In the CPS ≥10 group, 2 patients achieved CR, 6 patients achieved PR, 5 patients achieved SD, and 3 patients achieved PD. In the CPS < 10 group, 1 patient achieved CR, 3 patients achieved PR, 1 patient achieved SD, and 2 patients achieved PD. There was no statistically significant difference (*p* = 0.857). Regarding the non-assessment group, there were 2 cases of CR, 16 cases of PR, 8 cases of SD, and 5 cases of PD. In the 50% dose group, 1 patient achieved CR, 7 patients achieved PR, 2 patients achieved SD, 2 patients achieved PD. In the 70% dose group, 1 patient achieved CR, 1 patient achieved PR, 1 patient achieved SD. There was no statistically significant difference (*p* = 0.446). Thus, although there was a wide range of reductions in the dose of chemotherapy, no significant differences were observed in the therapeutic effect.

**Figure 1:**
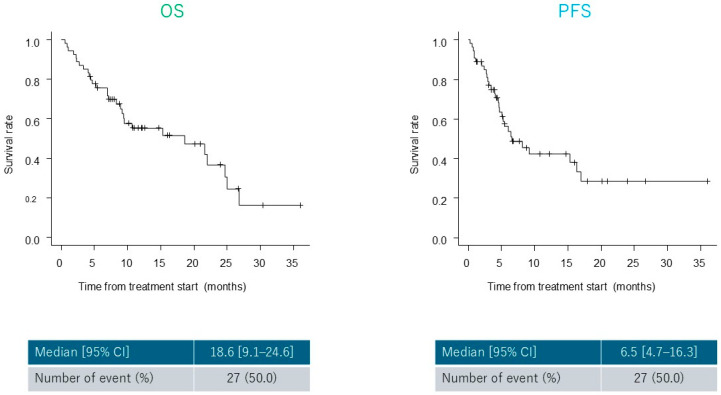
Survival outcomes. Kaplan–Meier curves for overall survival (OS) and progression-free survival (PFS) in all patients treated with cisplatin and 5-fluorouracil (CF) plus pembrolizumab (Pem) (CF plus Pem). The median OS and PFS were 18.6 (range, 9.1–24.6) and 6.5 (range, 4.7–16.3) months, respectively.

**Figure 2:**
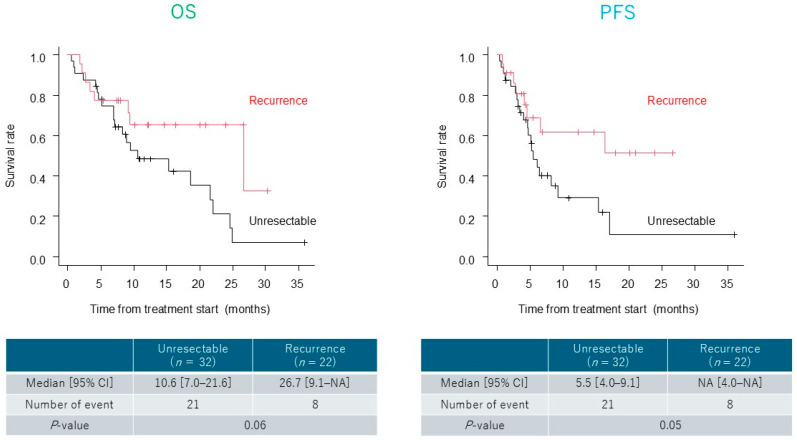
Survival outcomes stratified by reason for treatment. Comparison of overall survival (OS) and progression-free survival (PFS) between patients with unresectable and recurrent esophageal cancer. No significant difference in OS (*p* = 0.06) or PFS (*p* = 0.05) was observed between the two groups.

**Figure 3:**
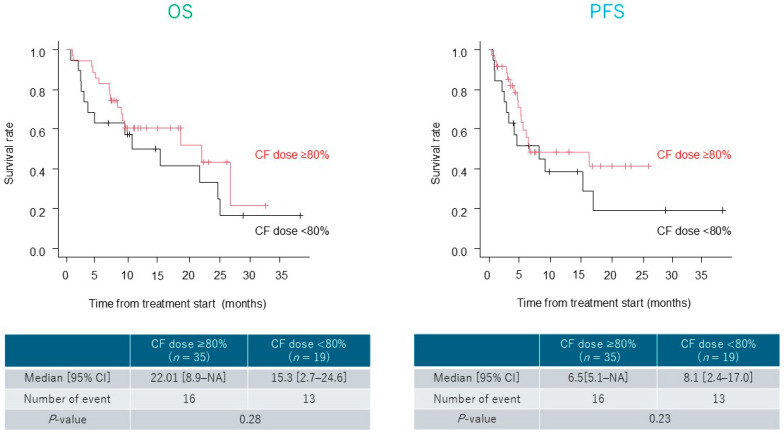
Survival outcomes stratified by the cisplatin and 5-fluorouracil dose (CF). Comparison of overall survival (OS) and progression-free survival (PFS) between the lower dose (initial CF dose < 80%) and higher dose (80–100%) groups. No significant difference in OS (*p* = 0.28) or PFS (*p* = 0.23) was observed between the two groups.

The median OS and PFS were 18.6 (range, 9.1–24.6 months) and 6.5 months (range, 4.7–16.3 months), respectively (Figure 1). Although not statistically significant, patients with recurrent disease showed a trend toward longer OS and PFS than those with unresectable disease (OS, *p* = 0.06; PFS, *p* = 0.05) (Figure 2). In the lower dose group (initial CF dose < 80%), the median OS and PFS were 15.3 (range, 2.7–24.6 months) and 8.1 months (range, 2.4–17.0 months), respectively. No significant differences in OS or PFS were observed between groups (OS, *p* = 0.28; PFS, *p* = 0.23) (Figure 3).

**Figure 4:**
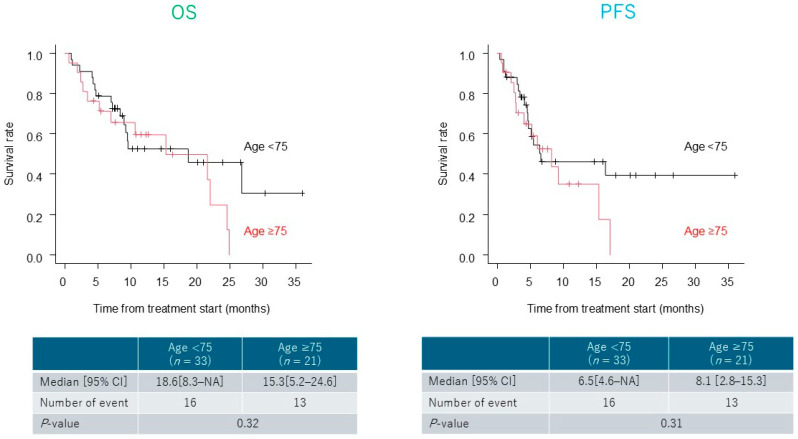
Survival stratified by age (≥75 vs. <75 years). Subgroup analysis of overall survival (OS) and progression-free survival (PFS) by age (≥75 vs. <75 years). No significant difference in OS or PFS was found in the subgroup analyses.

Survival analysis based on age (≥75 vs. <75 years) showed no significant differences in OS or PFS (OS, *p* = 0.32; PFS, *p* = 0.31) (Figure 4). Similarly, no significant differences in OS or PFS were observed between patients treated at the 3- and 4-week intervals.

### 3.4. Safety

Adverse events of grade 3 or higher were observed in 10 patients (18.5%). The most frequent adverse event was neutropenia (six cases), while immune-related adverse events included interstitial pneumonitis (one case) and autoimmune encephalitis (one case) (Table 4). Adverse events were compared between the ≥80% dose group (*n* = 35) and the <80% dose group (*n* = 19) (Table 5). Although some adverse events were observed only in the <80% dose group, no statistically significant differences were detected between the two groups (all *p*-value > 0.05).

In our study, hyponatremia occurred during the first cycle and was managed with intravenous supplementation; however, subsequent decline in performance status led to discontinuation of chemotherapy. Hypocalcemia and hypomagnesemia developed during the second cycle, both managed with intravenous supplementation, after which treatment was continued with dose reduction. Adrenal insufficiency was observed in one patient during the second cycle, requiring oral steroid therapy, with chemotherapy continuation. Two additional cases occurred during the fifth cycle: one was managed with oral steroids and treatment continued, while the other required steroid pulse therapy followed by oral steroids, resulting in treatment discontinuation. Hypothyroidism occurred in two patients (during the first and fifth cycles, respectively), both requiring initiation of oral medication. Autoimmune encephalitis developed in one patient during the fifth cycle, necessitating steroid pulse therapy and subsequent discontinuation of chemotherapy. Interstitial pneumonitis developed during the sixth cycle and was managed with steroid pulse therapy, also leading to discontinuation of chemotherapy. The incidence of grade 3 or higher adverse events was lower in the lower dose group than in the higher dose group, with only two cases reported.

## 4. Discussion

The advent of ICIs has substantially changed the treatment paradigm for advanced esophageal cancer. The KEYNOTE-590 trial demonstrated that CF plus Pem therapy significantly improved OS and PFS compared with CF plus placebo, particularly in patients with high PD-L1 expression [13]. However, this trial included patients with relatively favorable clinical profiles—such as younger age, preserved renal function, and good PS—raising concerns about the generalizability of its findings among older or comorbid populations, especially considering the nephrotoxicity of cisplatin-based regimens [14,15,16].

In this study, we retrospectively analyzed the real-world clinical outcomes of CF plus Pem therapy for unresectable or recurrent esophageal cancer. A notable feature of this analysis was the inclusion of patients who underwent initial dose reduction owing to advanced age, renal dysfunction, or poor PS. This study provides meaningful insights into the practical feasibility, effectiveness, and safety of this regimen in routine clinical practice. In our study, approximately 40% of patients were aged ≥75 years and approximately 45% had impaired renal function (eGFR < 60 mL/min/1.73 m^2^ or Ccr < 60 mL/min). Only 44.4% of patients received the full initial CF dose; the remainder underwent dose reduction primarily because of renal dysfunction or advanced age. Importantly, the ORR and DCR were comparable between the dose reduction and non-reduction groups 63.2% vs. 51.4% for ORR and 89.5% vs. 77.1% for DCR), with no significant differences (ORR: *p* = 0.57; DCR, *p* = 0.47). These results suggest that individualized dose adjustments, such as initial dose reduction based on age, renal function, and PS, may help preserve therapeutic efficacy, while potentially reducing the risk of treatment discontinuation due to toxicity, particularly in vulnerable populations [9,15]. The median OS and PFS were 18.6 (range, 9.1–24.6 months) and 6.5 months (range, 4.7–16.3 months), respectively. When stratified by disease status, patients with recurrent disease demonstrated a trend toward longer OS and PFS than those with initially unresectable disease, although the differences were not significant (OS, *p* = 0.06; PFS, *p* = 0.05). These nonsignificant trends may be attributed to the limited statistical power owing to the small sample size of this retrospective study. These differences may become more pronounced and reach statistical significance in larger cohorts. In the lower dose group (initial CF dose < 80%), the median OS and PFS were 15.3 (range, 2.7–24.6 months) and 8.1 months (range, 2.4–17.0 months), respectively, with no significant difference in OS or PFS (OS, *p* = 0.28; PFS, *p* = 0.23) compared to higher dose group. Notably, the median OS in the lower dose group was comparable with that reported in the KEYNOTE-590 trial (Pem plus chemotherapy median OS, 12.4 months in the overall population) [13]. This suggests that clinically meaningful survival benefits may be maintained in real-world settings even with an initial dose reduction. In older patients, a comprehensive assessment is essential because of age-related decline in organ function, comorbidities, and nutritional status. Aggressive treatment based solely on chronological age is often withheld; however, recent studies have suggested that personalized treatment based on tumor characteristics can yield favorable outcomes in older adults [16,17,18,19,20,21]. Our previous study reported good outcomes in patients aged ≥80 years who underwent dose-adjusted definitive chemoradiotherapy [22]. The present findings reinforce this notion, indicating that appropriate dose modifications can lead to satisfactory outcomes even in older patients. Furthermore, the incidence of grade ≥3 adverse events was relatively low (18.5%). Major adverse events included neutropenia, interstitial pneumonia, and irAEs, all of which were manageable with appropriate interventions. The lower incidence of irAEs in older patients may be due to immunosenescence, which reduces the immune reactivity. Some studies have suggested that irAEs may be less frequent in this population [23,24,25,26,27], supporting the rationale for administering Pem without dose reduction. Although this study included patients with advanced disease and distant metastases, some achieved CR or sustained SD, suggesting that the durable antitumor effects of immunotherapy may extend to metastatic lesions. Previous reports have shown better response rates in patients with lung or regional lymph node metastases, possibly because of the characteristics of the tumor microenvironment (TME) [28,29,30]. ICIs are thought to modify the TME, enhance immune cell infiltration, and improve tumor recognition, potentially leading to synergistic effects when combined with chemotherapy. These mechanisms may contribute to improved metastatic control and pave the way for conversion surgery, ultimately leading to improved survival.

Nonetheless, this study had some limitations that must be acknowledged. First, the retrospective, single-center nature of this study may have introduced selection and information biases. Second, the relatively small sample size limited the statistical power of multivariate and subgroup analyses. Thirdly, the relatively short follow-up period may have resulted in immature survival data. Future multicenter prospective studies are required to validate these findings and clarify the role of CF plus Pem therapy in real-world clinical settings.

## 5. Conclusions

Despite the limitations of this retrospective single-institution study, our findings provide important real-world evidence regarding the use of CF plus pembrolizumab for unresectable or recurrent esophageal cancer. The overall response rate was 55.6%, with a disease control rate of 81.5%, and the median progression-free and overall survival were 6.5 and 18.6 months, respectively. Notably, dose reduction of cisplatin and 5-fluorouracil, often required in elderly patients or those with renal dysfunction, did not compromise treatment efficacy, and the incidence of grade ≥3 adverse events was lower in the reduced-dose group. These results suggest that CF plus pembrolizumab can be safely and effectively administered across diverse clinical backgrounds, including older patients and those with impaired renal function. Given the aging global population, the implementation of flexible, patient-tailored treatment strategies is essential. This study highlights the feasibility of dose-adjusted CF plus pembrolizumab as a practical treatment option and provides valuable evidence to guide future optimization and personalization of therapy for patients with unresectable or recurrent esophageal cancer.

## Figures and Tables

**Table 1:** Characteristics of the patients.

Variable	Value
Age (median)	72.5 (44–91)
Sex (male/female)	40/14
Performance status (0–1)	41 (75.9%)
Renal function	
eGFR < 60	15 (27.3%)
Ccr < 60	26 (48.1%)
Ccr < 50	15 (27.8%)
With comorbidities	37 (68.5%)
Cardiovascular disorders	30 (55.6%)
Respiratory disorders	5 (9.3%)
Renal disorders	8 (14.8%)
Hepatic disorders	5 (9.3%)
Endocrine disorders	14 (25.9%)
Reason for treatment (unresectable/recurrent disease)	32/22
Tumor characteristics	
Location (Ut/Mt/Lt/Jz)	13/28/10/3
Histology (SCC/AD)	53/1
Macroscopic type (0/1/2/3/4)	4/5/14/26/5
T (1/2/3/4)	4/0/28/22
N (0/1/2/3)	7/19/15/13
M (0/1)	30/24
Stage (I/II/IIIA/IIIB/IVA/IVB) *	3/3/11/3/10/24
Metastatic sites	
Distant lymph nodes	15
Lung	7
Liver	7
Others	6
CPS	
≥10	16(69.6% **)
<10	7(30.4% **)
Not tested	31

CPS: combined positive score; Ut: upper thoracic esophagus; Mt: middle thoracic esophagus; Lt: lower thoracic esophagus; Jz: zone of the esophagogastric junction; SCC: squamous cell carcinoma; AD: adenocarcinoma. * Stage was classified according to the UICC 8th edition TNM classification at the time of initial diagnosis. For recurrent cases (*n* = 22), stage classification was based on the clinical stage before the initial therapy. ** Percentage of tested cases.

**Table 2:** Dose intensity of drugs and modification of the treatment interval.

Variable	Value
% Dose of Pembrolizumab (median)	100% (100)
% Dose of CF (median)	80% (50–100)
100%	24 (44.4%)
90%	2 (3.7%)
80%	9 (16.7%)
<80%	19 (35.2%)
Reason for reduction of CF	
Renal dysfunction	24 (44.4%)
Age	14 (26.0%)
PS ≥ 2	13 (24.1%)
Other	6 (11.1%)
Treatment cycle (3 weeks/4 weeks)	22 (40.7%)/27 (50.0%) *
Number of treatment course (median)	4 [1–23]
Post-treatment	30 (55.6%)
2nd-line chemotherapy	15
CRT	9
CS	5
RFA	1

CF: cisplatin plus 5-fluorouracil; PS: performance status; CRT: chemoradiotherapy; CS: conversion surgery; RFA: radiofrequency ablation. * Five patients underwent only one treatment course.

**Table 3:** Treatment outcomes.

Therapeutic Effect	Overall (*n* = 54)	Dose-Reduced (*n* = 19)	Age ≥ 75 (*n* = 21)
CR	5	2	3
PR	25	10	10
SD	14	5	6
PD	10	2	2
ORR	55.6%	63.2%	61.9%
DCR	81.5%	89.5%	90.5%

CR: complete response; PR: partial response; SD: stable disease; PD: progressive disease; ORR: overall response rate; DCR: disease control rate.

**Table 4:** Observed adverse events.

Adverse Events	Grade (CTCAE ver.5.0)	*n* (%) *
Anorexia	1/2/3	3/23/1 (50.0%)
Nausea	2	1 (1.9%)
Oral mucositis	2/3	2/1 (5.6%)
Creatinine increase	1/2/4	2/2/1 (9.3%)
Pneumonitis	2	1 (1.9%)
Leukopenia	2/3	2/3 (9.3%)
Neutropenia	2/3	2/6 (14.8%)
Febrile Neutropenia	3	1 (1.9%)
Platelet cell decrease	2	1 (1.9%)
Cerebral infarction	2	1 (1.9%)
Hyponatremia (irAE)	3	1 (1.9%)
Hypocalcemia (irAE)	2	1 (1.9%)
Hypomagnesemia (irAE)	2	1 (1.9%)
Adrenal insufficiency (irAE)	2	3 (5.6%)
Hypothyroidism (irAE)	2	2 (3.7%)
Autoimmune encephalitis (irAE)	2	1 (1.9%)
Interstitial pneumonia (irAE)	3	1 (1.9%)

CTCAE: common terminology criteria for adverse events; irAE: immune-related adverse event. * *n* (%) indicates the total number and percentage of patients experiencing each adverse event, regardless of grade.

**Table 5:** Adverse events according to dose intensity (≥80% vs. <80%).

Adverse Events	≥80% (*n* = 35)	<80% Dose (*n* = 19)	*p*-Value
Anorexia	17 (48.6%)	10 (52.6%)	1
Nausea	1 (2.9%)	0	1
Oral mucositis	2 (5.7%)	1 (5.3%)	1
Creatinine increase	5 (14.3%)	0	0.149
Pneumonitis	1 (2.9%)	0	1
Leukopenia	3 (8.6%)	2 (10.5%)	1
Neutropenia	6 (17.1%)	2 (10.5%)	0.698
Febrile Neutropenia	1 (2.9%)	0	1
Platelet cell decrease	1 (2.9%)	0	1
Cerebral infarction	0	1 (5.3%)	0.352
Hyponatremia (irAE)	0	1 (5.3%)	0.352
Hypocalcemia (irAE)	0	1 (5.3%)	0.352
Hypomagnesemia (irAE)	0	1 (5.3%)	0.352
Adrenal insufficiency (irAE)	1 (2.9%)	2 (10.5%)	0.28
Hypothyroidism (irAE)	2 (5.7%)	0	0.535
Autoimmune encephalitis (irAE)	1 (2.9%)	0	1
Interstitial pneumonia (irAE)	1 (2.9%)	0	1

irAE: immune-related adverse event.

## Data Availability

All data reported in this manuscript are available from the corresponding author upon reasonable request.
